# Naphthol in Dyspepsia

**Published:** 1890-03-22

**Authors:** 


### NAPHTHOL IN DYSPEPSIA.

Beta naphthol has a good reputation as an antiseptic for the alimentary canal. Some French physicians use it in the typhoid of children, three grains every hour, combined with an equal quantity of salicytate of bismuth, if there be diarrhœa, or salicylate of magnesium for diarrhœa. Dyspepsia dependent on ptomaines following stomach fermentation, may be treated according to this formula: R. Naphthol B. ʒiss, Bismuthi Salicylates ʒiij, M. et div. in capsulis No. XXX.; sign, one capsule with each meal. Magnesia may be added, if necessary, to this prescription. The *rationale* of the treatment is to check fermentation, and to neutralise toxic products.

